# Health, Suicidal Thoughts, and the Life Course: How Worsening Health Emerges as a Determinant of Suicide Ideation in Early Adulthood

**DOI:** 10.1177/00221465221143768

**Published:** 2023-01-12

**Authors:** Carlyn Graham, Andrew Fenelon

**Affiliations:** 1 The Pennsylvania State University - University Park Campus, University Park, PA, USA

**Keywords:** early adulthood, life course, self-rated health, suicide ideation

## Abstract

Poor physical health places adults at greater risk for suicide ideation. However, the linkage between health and suicidal thoughts may emerge and become established during early adulthood, concomitant with other social processes underlying suicidality. Using nationally representative survey data from Waves III through V of the National Longitudinal Study of Adolescent to Adult Health (n = 8,331), we examine the emergence of health as a predictor of suicide ideation across the early adult life course (ages 18–43). We find that worsening health does not significantly predict suicide ideation until young adults approach the transition into midlife. Our findings suggest this may be due to the increasing severity of health problems, reduced social network engagement, and disruption of social responsibilities later in early adulthood. Our findings underscore the need for social science research to examine the relationship between mental and physical health from a life course perspective.

Suicide is one of five leading causes of death among 15- to 44-year-olds in the United States (NIMH 2022). As of 2020, adults ages 18 to 25 have the highest prevalence of suicidal thoughts of any adult age group, followed by adults ages 26 to 49 (National Institute of Mental Health [NIMH] 2022). While most individuals with suicide ideation do not attempt suicide (Klonsky, May, and Saffer 2016), the majority of suicide attempters and completers experience suicide ideation at some point during their lives (McAuliffe 2002). As such, identifying risk factors for suicide ideation is a critical component of suicide prevention (Chang et al. 2021).

Early adulthood is a life course period characterized by significant life transitions, such as leaving the parental home, pursuing higher education, entering the workforce, and forming unions and households (Bültmann et al. 2020). During these transitions, disruptive experiences such as separation or job loss can lead to increased social isolation (Eckhard 2018) and raise the risk of suicidal thoughts (Calati et al. 2019; Gratz et al. 2020). Specifically, poor physical health might increasingly impede the social roles, responsibilities, and relationships of younger adults, yet research has not considered how the relationship between poor health and suicide ideation develops throughout early adulthood.

Among adults, poor physical health, such as having chronic conditions, can contribute to social isolation (Hawthorne 2008), difficulty sustaining close relationships (Haas, Schaefer, and Kornienko 2010), and the diminished capacity to fulfill social roles and responsibilities, including caring for children (Wolf, Freisthler, and McCarthy 2021) and maintaining employment (Antonisse and Garfield 2018). Subsequently, social isolation and disruption of social roles and responsibilities are leading risk factors for suicide ideation (Calati et al. 2019). For example, loss of employment can lead to psychosocial distress, loss of social network connections, and non-work-based relationship strain (Fiksenbaum et al. 2017). Unsurprisingly, poor self-rated physical health and having chronic conditions can heighten the risk of suicide ideation among adults (Goodwin and Marusic 2011; Goodwin and Olfson 2002; Lutz et al. 2016).

We may expect poor physical health to emerge as an important predictor of suicidal thoughts in early adulthood for several reasons. First, health events, such as hospitalizations or the onset of chronic conditions, may grow in severity as individuals age. For example, health problems earlier in adulthood may be relatively minor, while later in adulthood, the emergence of chronic conditions could have a stronger negative effect on quality of life and mental health (Heyworth et al. 2009). Second, the effect of poor health on social network engagement may become increasingly consequential for suicide ideation. By their late 30s, adults have made substantial investments in relationships, making them especially vulnerable to threats to these bonds (Shiner et al. 2009). Poor physical health can weaken close relationships, which has implications for feelings of social isolation. Finally, poor health may emerge as a predictor of suicidal thoughts later in early adulthood because adults increasingly take on roles and responsibilities throughout early adulthood; as they enter into marriage, parenthood, and long-term employment, poor physical health may reduce the ability to maintain these responsibilities.

We employ a nationally representative longitudinal survey to examine the life course emergence of health as a predictor of suicidal ideation during early adulthood (ages 18–45; Levinson 1986). Specifically, we distinguish between two periods, “emerging adulthood” (approximately 18–29) and “young adulthood” (approximately 30–45; Arnett 2012, 2019). We exploit within-person changes in self-reported health status across Waves III through V of the National Longitudinal Study of Adolescent to Adult Health (Add Health) to investigate worsening health as a predictor of suicidal ideation across early adulthood net of sociodemographic characteristics, life transitions, and mental health. We find that while worsening health is not associated with suicide ideation in emerging adulthood, worsening health emerges as a significant predictor of suicide ideation during young adulthood. We test several potential explanations for the increasing importance of health as a predictor of suicide ideation, including health severity, effect of worsening physical health on social network engagement, and effect of worsening health on social roles and responsibilities. Our findings emphasize the importance of a life course perspective in examining suicide ideation and contextualizing health experiences among emerging and young adults within their rapidly changing lives.

## Background

### Life Course Processes and Suicide

Sociologists have an interest in a life course perspective because long-term sequences of events in various age-graded realms can be used to link scholarship on multiple domains of phenomena. The life course presents the opportunity to contextualize complex social relationships between processes that would seem disconnected without a longer-range view.

Sociologists define life course patterns as the sequence of states and events in realms of life that span from birth to death (Diewald and Mayer 2009). Life course trajectories include various life transitions (including the transition to adulthood; Denice 2019), and while the transition into adulthood increasingly marks a period of demographic variability (Eliason, Mortimer, and Vuolo 2015), many adults follow normative life course patterns in early adulthood (Amato and Kane 2011). For example, most adults leave their parental homes to pursue higher education or employment and eventually transition into cohabitation, marriage, parenthood, and/or long-term employment (Arnett 2019; Eliason et al. 2015).

Arnett (2019) argues that “emerging adulthood” roughly encapsulates ages 18 to 29 and represents a distinct period in the life course characterized by identity exploration and self-focus through opportunities to traverse education, work, place of residence, and relationships. Following adolescence, most emerging adults leave home and become more independent of their parents but have yet to enter into enduring roles and commitments through long-term employment, marriage, and parenthood (Arnett 2019). Consequently, emerging adulthood is marked by instability and transience in various domains of life (Arnett 2019). For example, emerging adults ages 20 to 29 change jobs on average seven times, and relationships are often temporary during this phase of the life course (Arnett 2012). Of note, albeit distinct in many ways, emerging adulthood has also been described as an “extended adolescence” insofar as both adolescence and emerging adulthood are imbued with identity and relationship exploration (Arnett 2019).

Following developmental milestones that often characterize emerging adulthood, emerging adults transition into “young adulthood,” a period Arnett (2012) defines as the early 30s to mid-40s (roughly 30–45) that is marked with greater stability and more permanent roles, responsibilities, and relationships. More so today than ever in the United States, young adults pursue careers that require a postsecondary education due in large part to the increasing economic returns to higher education (Settersten, Ottusch, and Schneider 2015; Tillman, Brewster, and Holway 2019). Consequently, many emerging adults delay marriage and parenthood until young adulthood, after completing education and/or securing stable employment (Settersten et al. 2015; Zapata-Gietl et al. 2016). As a result, these emerging adults have also postponed the roles and responsibilities that accompany marriage, parenthood, and long-term employment (Zapata-Gietl et al. 2016). Overall, these life course trajectories have implications for both physical and mental health.

From a life course perspective, factors that contribute to suicide risk evolve alongside various life transitions (Brenner and Barnes 2012; Cohler and Jenuwine 1995; Shiner et al. 2009) that may be especially pronounced in early adulthood. Scholars of the sociology of suicide underscore the protective nature of social roles and bonds, particularly through work and family life, because they provide people with a sense of purpose and belonging (Durkheim [1897] 1951; Mueller and Abrutyn 2016; Mueller et al. 2021). Concomitantly, disintegration of social roles and bonds through disruptions such as divorce or loss of employment can lead to social isolation, status loss, and threats to identity that contribute to suicide (Abrutyn 2019; Mueller et al. 2021).

However, the role of social roles, responsibilities, and bonds may not emerge as the most salient suicidal risk factors until young adulthood. For example, predominant suicidal risk factors among emerging adults under the age of 25 are not necessarily threats to social roles or bonds but, rather, factors such as childhood abuse or neglect (Shiner et al. 2009). As young adults approach midlife, risk factors for suicide evolve to include disruptions of social roles, responsibilities, and bonds, such as employment instability and family breakdown (Gratz et al. 2020; Kyung-Sook et al. 2018; Shiner et al. 2009). As Shiner et al. (2009) argue, young people (under age 30) are not immune to the emotional consequences of relationship breakdown, but they tend to be less invested in the permanence of their relationships than older adults, and thus, relationship breakdown may not contribute to suicide risk until later adulthood. Based on this perspective, we argue it is pertinent to examine the relationship between physical health and suicidality from a life course perspective, especially throughout early adulthood, a period with substantial developments in the nature of social roles, responsibilities, and bonds.

### Physical Health and Suicidality

Extant literature has explored the relationship between physical health and suicidality among adults. This research indicates that having chronic conditions, such as asthma and diabetes, increases the likelihood of suicide ideation (Barker, Kãlves, and De Leo 2015; Lutz et al. 2016). Furthermore, among representative samples of U.S. adults, self-perceived poor health has been linked to augmented risk of suicidal ideation, net of sociodemographic characteristics, mental disorders, and physical illnesses (Goodwin and Marusic 2011; Goodwin and Olfson 2002). However, a paucity of literature exists on the relationship between self-reported health and suicide ideation within the last decade, and none, to our knowledge, has examined this relationship throughout early adulthood.

One study has examined self-reported health and suicidality explicitly among younger adults in the United States. Utilizing a nationally representative sample of adults ages 17 to 39, Zhang et al. (2005) found that women with “poor” self-perceived health had higher odds of suicide attempts relative to women with “excellent” self-perceived health. However, given the cross-sectional nature of the data, the authors could not capture the relationship between self-reported health and suicidality as adults move through early adulthood. Our study expands on these findings by assessing the association between changes in self-reported health and suicide ideation throughout early adulthood.

### Health, the Life Course, and Suicidal Ideation

Health is an important aspect of well-being, and its role as a primary determinant of quality of life grows across the life course (Plagnol and Scott 2011). Similarly, the importance of health as a predictor of suicide ideation may grow across the early adult life course for several reasons. Specifically, health may emerge as an increasingly important predictor of suicide ideation because (a) the severity of health problems increases with age, (b) the importance of social ties increases in young adulthood and poor health limits social engagement, and (c) health problems disrupt social roles and responsibilities that young adults increasingly take on and that subsequently become more demanding as they age into their 30s and 40s.

*Hypothesis 1:* Worsening health will have a larger effect on suicide ideation in young adulthood relative to emerging adulthood.

### Severity of Health Problems across the Life Course

First, health problems in young adulthood may be more severe than those experienced during emerging adulthood. While health tends to decline monotonically with age even in early adulthood (Sokol et al. 2017), there are also significant age-graded patterns in the disruptiveness of health events that adults experience. Specifically, health events may increasingly transition from acute events (e.g., illnesses or injuries) to chronic conditions (e.g., cardiovascular or metabolic conditions), which have a more persistent negative effect on life satisfaction (Strine et al. 2008). The increasing age-related prevalence of chronic diseases may indicate that health issues later in adulthood are likely to have a more significant negative impact on quality of life and play a more pronounced role in individuals’ day-to-day experiences (Kane et al. 2018).

Self-rated health has been validated as reliable indicator of objective health (Wu et al. 2013), and irrespective of age, evidence suggests that perceived health heavily reflects underlying disease burden (Kaplan et al. 1996). In this case, we would expect that worsening health will reflect a greater severity of health problems in young adulthood relative to emerging adulthood.

*Hypothesis 2:* Worsening health will predict more chronic conditions in young adulthood than emerging adulthood.

### Health and Social Network Engagement

Second, the effect of poor health on social network engagement may be more consequential for suicide ideation during young adulthood than emerging adulthood. Poor physical health diminishes the likelihood of social engagement (Rochelle and Shardlow 2014), subsequently leading to increased feelings of social isolation (Hawthorne 2008). For example, poor health generates stressors and decreases the amount of energy that can be committed to relationships, which may reduce the ability to sustain them (Haas et al. 2010). Evidence suggests that social network ties may increase in importance for suicide ideation throughout early adulthood. For example, emerging adulthood is a period marked with individualism and identity exploration (Arnett 2019), and consequently, relationships such as friendships and romantic partners are often temporary and unstable (Arnett 2012).

As emerging adults enter young adulthood, they become embedded in larger and more permanent social networks (Harris and McDade 2018). Furthermore, while the number of friends in social networks tends to decline with age in adulthood, these decreases reflect greater investment in satisfying and emotionally close relationships (Rook and Charles 2017). Indeed, socioemotional selectivity theory suggests that younger people prioritize exploring and acquiring new relationships, which is especially salient in emerging adulthood, whereas older adults favor deepening established intimate relationships (Sullivan-Singh, Stanton, and Low 2015). In other words, younger people are less socially embedded than their older counterparts, and by the mid-to-late 30s, adults have invested substantially in their relationships, making threats to these bonds especially detrimental (Shiner et al. 2009). Moreover, close network ties are more protective against loneliness and isolation for older adults than younger adults (Child and Lawton 2019). Thus, particularly because social network ties are more significant and permanent in young adulthood than emerging adulthood, the deleterious effect of disruption of social network engagement due to poor physical health may become more robust with age.

*Hypothesis 3a:* Worsening health will disrupt close friendship ties.
*Hypothesis 3b:* The number of close friends will have a stronger relationship to suicide ideation in young adulthood relative to emerging adulthood.

### Health and Disruption of Social Roles and Responsibilities

Finally, physical health may grow in importance because young adults increasingly take on more permanent responsibilities and social roles as they age that can be disrupted by poor health. For example, most emerging adults have not entered into stable and enduring adult roles, such as long-term employee, spouse, and parent, and acquired the concomitant responsibilities (Arnett 2019). By the 30s and 40s, young adults have largely entered into these established roles that entail daily requirements and obligations (Arnett 2012). Furthermore, social roles and pressures, such as sustaining a career, are likely to become more demanding in young adulthood (Umberson, Crosnoe, and Reczek 2010). For example, jobs during the emerging adult years are often temporary or part-time, and not until the early 30s do young adults typically enter more stable long-term jobs (Arnett 2012, 2019). Once young adults enter into more permanent jobs, the role requirements increase because these jobs are likely connected to long-term career paths (Arnett 2012, 2019). As such, the role demands in young adulthood are often the most substantial out of any phase in the life course (Arnett 2012).

Poor health inhibits the ability to fulfill family responsibilities (Percheski and Meyer 2018), such as caring for children (Wolf et al. 2021), performing marital roles (Caputo and Simon 2013), and maintaining employment (Antonisse and Garfield 2018). Furthermore, disruption of roles and statuses, including the relationships and activities associated with them, often occur during later phases of the life course (Pearlin et al. 2005). For example, among married parents, fathers’ health-related work limitations and mothers’ poor self-rated health contribute to marital dissolution (Percheski and Meyer 2018). Experiencing poor health may increase stress and the risk of suicide ideation if they prevent young adults from fully participating in their work and family duties.

*Hypothesis 4a:* Worsening health will have a larger effect on suicide ideation for those who are more employed than those who are less employed. This pattern will be especially pronounced in young adulthood given the increasing investments into employment with age.
*Hypothesis 4b:* Worsening health will have a larger effect on suicide ideation for those with children in the household than those without children in the household throughout early adulthood. Given the increasing fraction of adults with children in young adulthood relative to emerging adulthood, this could potentially lend support for Hypothesis 1.
*Hypothesis 4c:* Worsening health will have a larger effect on suicide ideation among the married than the unmarried throughout early adulthood. Given the increasing fraction of married adults in young adulthood relative to emerging adulthood, this could potentially lend support for Hypothesis 1.

## Data and Methods

### Data

We used data from the National Longitudinal Study of Adolescent to Adult Health (Add Health), a longitudinal study of adolescents in Grades 7 to 12 in 1994 to 1995. Five waves of data were collected between 1994 to 1995 and 2016 to 2018. Wave I included 90,118 students from 145 middle, junior, and high schools, and 20,745 of them were selected to complete the 1994 to 1995 in-home interview. Wave II data were collected in 1996 and included 14,738 adolescents. Wave III data were collected in 2001 to 2002, when the Wave I participants were emerging adults ages 18 to 26 (n = 15,197 respondents). Waves IV and V were collected in 2008 and 2016 to 2018, respectively. Respondents were ages 24 to 32 in Wave IV (n = 15,701) and ages 33 to 43 in Wave V (n = 12,300). There were 9,349 respondents of the Wave I sample interviewed across Waves III, IV, and V.

The target population of our study was emerging and young adults, and therefore, we used data from Waves III to V, when the respondents were ages 18 to 43. Waves III and IV captured emerging adulthood (18–32), and Wave V captured young adulthood (33–43). We also used demographic data from Wave I such as sex and race-ethnicity. We used survey weights, and the longitudinal survey weight for Waves III through V included Wave I respondents interviewed at Waves III, IV, and V. Our weighted analyses excluded 170 respondents due to missing U.S. region. We employ listwise deletion to handle missing data, reducing our weighted analytic sample size to 8,331.

#### Outcome measure

Our outcome of interest was suicide ideation, based on the question, “During the past 12 months, have you ever seriously thought about committing suicide?” The response was dichotomous and coded as 0 (no) and 1 (yes).

#### Primary predictor

Our main predictor was *changes in health status*, based on the question, “In general, how is your health?” Responses included (1) excellent, (2) very good, (3) good, (4) fair, and (5) poor. We combined excellent and very good and fair and poor to create a three-category variable: (1) *excellent/very good*, (2) *good*, and (3) *fair/poor* (Zhang et al. 2005). Combining the fair and poor categories is typical (e.g., Goodwin and Marusic 2011; Lopez 2004) and maintains statistical power. Self-rated health is a reliable indicator of objective health status (Wu et al. 2013) and an independent predictor of mortality (Idler and Benyamini 1997).

We measured within-person *changes in health status* over time between Waves III through IV and Waves IV through V. Our categories included health remained the same (consistent), worsened, and improved. The consistent category (reference) included no change in health (e.g., excellent/very good in both waves), the worsened category included a decrease in health (e.g., good in Wave III to fair/poor in Wave IV), and the improved category included an improvement in health (e.g., good in Wave III to excellent/very good in Wave IV).

#### Controls

We included a series of sociodemographic control variables. Basic demographics included *sex* (0 = male, 1 = female), *race-ethnicity* (non-Hispanic White [reference], Hispanic, non-Hispanic Black, and non-Hispanic other), *nativity* (0 = foreign-born, 1 = U.S. born), and *age* (in years). We included *educational attainment* as a measure of socioeconomic status (SES), a widely used measure for SES when analyzing the relationship between SES and health (Shavers 2007; less than high school degree [reference], high school degree or GED, some college, and four-year degree or more).

Next, we included changes in marital status, employment status, and depression between Waves III through IV and IV through V, which could potentially confound the relationship between changes in health status and suicide ideation. For example, singlehood and divorce and unemployment and job loss heighten the risk of suicide ideation (Dalglish et al. 2015; Gratz et al. 2020; Kyung-Sook et al. 2018). We chose to include these predictors because they represent the major life transitions experienced throughout early adulthood. Furthermore, we included changes in depression in accordance with other studies examining the relationship between physical health and suicide ideation (Barker et al. 2015; Chung, Kim, and Lee 2016; Goodwin and Marusic 2011; Goodwin and Olfson 2002; Lutz et al. 2016).

We coded *changes in marital status* as consistently unmarried (reference), consistently married, transitioned into marriage, and transitioned out of marriage. We coded *changes in employment status* as consistently not employed ≥10 hours/week (reference), consistently employed ≥10 hours/week, transitioned into employment ≥10 hours/week, and transitioned out of employment ≥10 hours/week. Due to the employment status measure in the Add Health questionnaires in Waves III and IV (“Are you currently working for pay for at least 10 hours a week?”), we could not distinguish between unemployed and employed. We also included those active in the military as employed ≥10 hours/week.

We coded *changes in depression* as never depressed (reference), consistently depressed, depression onset, and depression recovery. We created the depression variable in each wave based on a modified nine-item (Waves III and IV) and five-item (Wave V) Center for Epidemiological Studies (CES-D) instrument. When response items were summed (ranges = 0–27 in Wave III and Wave IV and 0–15 in Wave V), higher scores were indicative of depression. In accordance with previous research, a score of 11 or greater in Waves III and IV (Frisco, Houle, and Lippert 2013) indicates depression. A cutoff point of 20 is adequate for the full CES-D scale (Vilagut et al. 2016), and for shortened versions of the CES-D, the cut point can be adjusted by multiplying it by n/20, where n is the number of items in the shortened scale (Lewinsohn et al. 1997). Therefore, we determined a score of 5 or greater in Wave V classified a respondent as having depression (1 = yes). Studies have supported the reliability and validity of the five-item subset of the CES-D (Bohannon, Maljanian, and Goethe 2003; Lewinsohn et al. 1997).

We then created a continuous variable for the number of *chronic conditions* that included the diagnosis of “cancer,” “high blood cholesterol,” “high blood pressure,” “diabetes,” and “asthma,” which were collected across Waves III through V. The number of *close friends* was a continuous measure with a range of 0 to 6. Finally, we created a variable for the *presence of children in the household* (0 = no children in the household, 1 = children in the household).

### Analytic Strategy

We used logistic regression to estimate the association between changes in health status and suicide ideation in two between-wave periods. The first set of models predicted suicide ideation in Wave IV (emerging adulthood) as a function of change in health status between Waves III through IV, and the second set of models did the same between Waves IV through V (young adulthood). As a reminder, respondents were between the ages of 24 and 32 in Wave IV and ages 33 and 43 in Wave V. For each period, we estimated the unadjusted relationship between changes in health status and suicide ideation. Next, we adjusted for sociodemographic controls. Finally, our fully adjusted model incorporated life transitions and changes in depression.

We tested three possible explanations for the emerging relationship between changes in health status and suicide ideation, including the severity of health conditions, disruption of social network engagement, and disruptions in social roles. To test if the severity of worsening health changes between waves, we estimated the association between changes in health status and the number of chronic health conditions in Waves IV and V using ordinary least squares (OLS) regression models adjusted for sociodemographic and life transitions/depression. Next, we determined if worsening health affects the number of close friends by estimating the association between changes in health status and the number of close friends in Waves IV and V using OLS regression models adjusted for sociodemographic and life transitions/depression. Then we controlled for the number of close friends in our fully adjusted main models to determine if the number of close friends had an effect on suicide ideation. Finally, we determined whether the effect of worsening health was a reflection of social roles and responsibilities by stratifying our main models by employment status, presence of children in the household, and then marital status.

## Results

### Descriptive Statistics

We present descriptive statistics of the Add Health sample in Table 1. Approximately half the sample is female (50.1%), and the majority identify as non-Hispanic White (65.6%). The mean age of respondents in Wave IV is 28.9 and 37.8 in Wave V. The highest level of educational attainment for the largest proportion of respondents in both waves is some college (43.4% and 41.1%).

**Table 1.** Weighted Descriptive Statistics for Waves IV and V (*n* = 8,331).

	Wave IV	Wave V
	% or Mean (SE)	% or Mean (SE)
Suicide ideation (1 = yes)	6.87	7.35
Changes in health status		
Consistent health (reference)	62.68	59.26
Worsened health	25.92	25.51
Improved health	11.40	15.23
Female (1 = yes)	50.05	50.05
Race-ethnicity		
Non-Hispanic White (reference)	65.63	65.63
Hispanic	11.84	11.84
Non-Hispanic Black	14.78	14.78
Non-Hispanic other	7.74	7.74
U.S. born (1 = yes)	94.52	94.52
Age	28.94 (.12)	37.83 (.12)
Educational attainment		
Less than high school (reference)	7.29	4.92
High school or GED	16.38	16.23
Some college	43.40	41.07
4-year degree or more	32.93	37.77
Changes in employment status		
Consistently not employed ≥10 hours/week (reference)	7.47	7.56
Consistently employed at ≥10 hours/week	63.18	73.49
Transitioned into employment ≥10 hours/week	20.45	8.81
Transitioned out of employment ≥10 hours/week	8.90	10.15
Changes in marital status		
Consistently unmarried (reference)	52.58	33.14
Consistently married	14.49	34.66
Transitioned into marriage	29.96	22.42
Transitioned out of marriage	2.98	9.78
Changes in depression		
Never depressed (reference)	73.46	71.46
Consistently depressed	6.21	6.73
Depression onset	12.02	10.31
Depression recovery	8.31	11.51
Number of chronic conditions	.38 (.01)	.62 (.02)
Number of close friends	3.93 (.05)	3.12 (.04)
Has children in the household (1 = yes)	46.81	68.58

*Source*: National Longitudinal Study of Adolescent to Adult Health (Add Health).

Approximately 7% of the sample in both Waves IV and V indicated that they had suicide ideation in the past 12 months. In both waves, the majority of respondents had consistent health (62.7% and 59.3%, respectively). A similar proportion of the sample had worsened health in each wave interval (26%). A larger proportion of respondents in Wave V were consistently employed (73.5%) than in Wave IV (63.2%). Furthermore, the majority of respondents in Wave V remained married or transitioned into marriage (57.1%), whereas the majority of respondents in Wave IV remained unmarried (52.6%). Finally, relative to Wave IV, respondents in Wave V had a larger mean number of chronic conditions (.62 vs. .38) and a smaller mean number of close friends (3.12 vs. 3.93), and a larger proportion (68.6% vs. 46.8%) had children in the household.

### Emergence of Health as a Predictor of Suicide Ideation

Table 2 presents logistic regression models predicting the odds of suicide ideation in Wave IV as a function of changes in health status between Waves III through IV. Compared with consistent health, worsened health significantly predicts suicide ideation (odds ratio [OR] = 1.50, 95% confidence interval [CI]: 1.11 to 2.03) in the unadjusted model. However, after adjusting for life transitions and depression in Model 3, worsened health no longer predicts suicide ideation (OR = 1.14, 95% CI: .84 to 1.54).

**Table 2.** Weighted Estimates (Odds Ratios) from Logistic Regression Models Predicting Suicide Ideation in Wave IV (*n* = 8,331).

	Model 1		Model 2		Model 3	
	OR	95% CI	OR	95% CI	OR	95% CI
Health status (reference = consistent)						
Worsened	1.50**	(1.11, 2.03)	1.37*	(1.02, 1.87)	1.14	(.84, 1.54)
Improved	1.23	(.84, 1.80)	1.15	(.78, 1.68)	.89	(.60, 1.31)
Female (reference = male)			1.14	(.84, 1.56)	.84	(.62, 1.14)
Race-ethnicity (reference = non-Hispanic White)						
Hispanic			.66*	(.45, .98)	.67^+^	(.45, 1.01)
Non-Hispanic Black			1.06	(.73, 1.52)	.85	(.56, 1.30)
Non-Hispanic other			1.12	(.68, 1.85)	.98	(.58, 1.66)
U.S. born (reference = foreign-born)			.81	(.45, 1.44)	.86	(.48, 1.53)
Age			.97	(.90, 1.04)	.96	(.88, 1.04)
Educational attainment (reference = less than high school)						
High school or GED			.87	(.55, 1.40)	1.23	(.75, 2.00)
Some college			.73	(.50, 1.09)	1.16	(.74, 1.80)
4-year degree or more			.49**	(.31, .76)	1.03	(.61, 1.76)
Employment status (reference = consistently not employed ≥10 hours/week)						
Consistently employed ≥10 hours/week					.56*	(.36, .87)
Transitioned into employment ≥10 hours/week					.50*	(.29, .85)
Transitioned out of employment ≥10 hours/week					.80	(.42, 1.53)
Marital status (reference = consistently unmarried)						
Consistently married					.90	(.55, 1.48)
Transitioned into marriage					.76^+^	(.56, 1.02)
Transitioned out of marriage					.65	(.31, 1.38)
Depression (reference = never depressed)						
Consistently depressed					10.28***	(7.25, 14.57)
Depression onset					5.94***	(4.50, 7.83)
Depression recovery					1.80^+^	(.99, 3.28)

*Source*: National Longitudinal Study of Adolescent to Adult Health (Add Health).

*Note*: OR = odds ratio; CI = confidence interval.

+ *p* < .1, *p < .05, **p < .01, ***p < .001.

The models in Table 3 predict suicide ideation in Wave V as a function of changes in health between Waves IV through V. Contrasting the results in Table 2, worsened health is associated with significantly higher odds of suicide ideation (OR = 1.52, 95% CI: 1.20 to 1.92) than consistent health in the fully adjusted model. Results in Appendix Table A1 in the online version of the article show that results are consistent when using chronic conditions as a measure of health. We also estimated our main models stratified by gender and did not find gender differences (Appendix Table D1 in the online version of the article).

**Table 3.** Weighted Estimates (Odds Ratios) from Logistic Regression Models Predicting Suicide Ideation in Wave V (*n* = 8,331).

	Model 1		Model 2		Model 3	
	OR	95% CI	OR	95% CI	OR	95% CI
Health status (reference = consistent)						
Worsened	2.12***	(1.71, 2.63)	2.03***	(1.62, 2.53)	1.52**	(1.20, 1.92)
Improved	.93	(.65, 1.33)	.88	(.61, 1.26)	.82	(.56, 1.21)
Female (reference = male)			.94	(.74, 1.20)	.75*	(.57, .99)
Race-ethnicity (reference = non-Hispanic White)						
Hispanic			.95	(.65, 1.38)	.84	(.57, 1.23)
Non-Hispanic Black			.73^+^	(.53, 1.01)	.52***	(.36, .74)
Non-Hispanic other			1.20	(.76, 1.91)	.89	(.56, 1.41)
U.S. born (reference = foreign-born)			.99	(.56, 1.74)	.67	(.39, 1.16)
Age			.92*	(.86, .99)	.89**	(.83, 096)
Educational attainment (reference = less than high school)						
High school or GED			.48**	(.28, .82)	.67	(.39, 1.16)
Some college			.56*	(.36, .87)	.84	(.52, 1.35)
4-year degree or more			.35***	(.22, .56)	.73	(.46, 1.18)
Employment status (reference = consistently not employed ≥10 hours/week)						
Consistently employed ≥10 hours/week					1.33	(.75, 2.37)
Transitioned into employment ≥10 hours/week					2.26*	(1.14, 4.49)
Transitioned out of employment ≥10 hours/week					2.14*	(1.11, 4.10)
Marital status (reference = consistently unmarried)						
Consistently married					.50***	(.35, .71)
Transitioned into marriage					.49***	(.34, .71)
Transitioned out of marriage					1.01	(.69, 1.46)
Depression (reference = never depressed)						
Consistently depressed					18.94***	(13.03, 27.54)
Depression onset					11.35***	(7.92, 16.25)
Depression recovery					2.63***	(1.65, 4.19)

*Source*: National Longitudinal Study of Adolescent to Adult Health (Add Health).

*Note*: OR = odds ratio; CI = confidence interval.

+ *p* < .1, *p < .05, **p < .01, ***p < .001.

**Figure 1.**
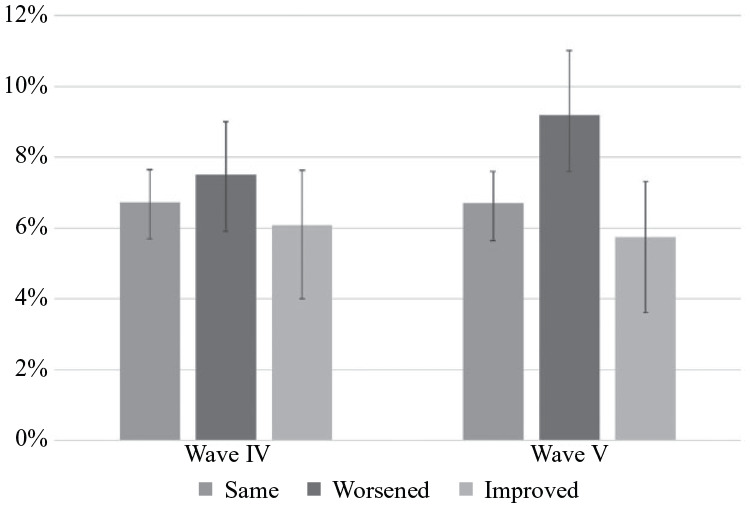
Predicted Probability of Suicide Ideation by Changes in Health Status Waves IV and V (*n* = 8,331). *Source*: National Longitudinal Study of Adolescent to Adult Health (Add Health).

The emergence of worsened health as a predictor of suicide ideation is shown graphically in Figure 1. Worsened health has the highest predicted probability of suicide ideation in Wave IV, although it is not significantly greater than consistent health. By Wave V, the importance of health emerges clearly: Individuals with worsened health have a significantly higher probability of suicide ideation compared to those with consistent health (9.2% vs 6.7%).

### Explaining the Increasing Importance of Health

**Figure 2.**
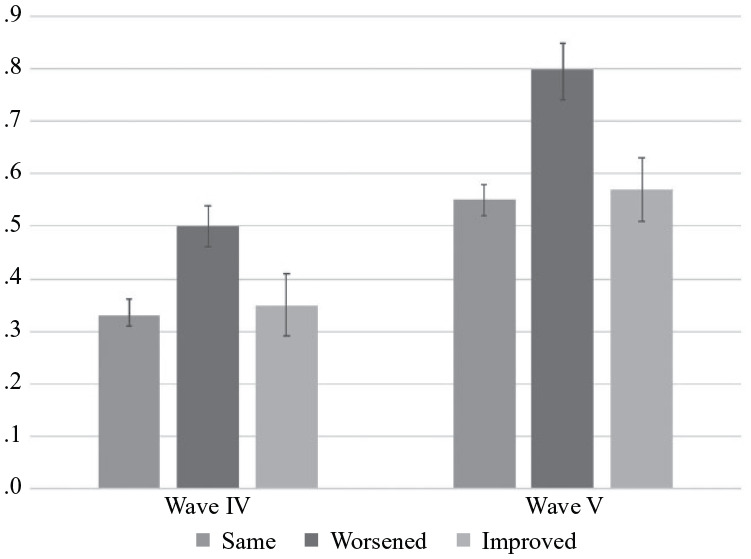
Predicted Number of Chronic Conditions by Changes in Health Status in Waves IV and V (*n* = 8,331). *Source*: National Longitudinal Study of Adolescent to Adult Health (Add Health).

We test three possible explanations for the emerging importance of health as a predictor of suicide ideation. First, we examine whether the increasing importance of health may reflect the rising severity of health problems. Figure 2 presents differences in predicted numbers of chronic conditions by health status in each wave. For Wave IV, worsened health is associated with .17 more chronic conditions than consistent health. In Wave V, this difference increases to .25, suggesting that health problems may be increasingly severe. Table A2 in the Appendix in the online version of the article demonstrates that ORs are attenuated after adjusting for chronic conditions.

Next, we examine whether worsened health predicts the number of close friends. Table 4 shows worsened health is associated with .11 fewer friends in Wave IV and .18 fewer friends in Wave V than consistent health. Then, we determine if the number of close friends is a significant predictor of suicide ideation. Table 5 shows that each unit increase in the number of close friends reduces the odds of suicide ideation by 15% in Wave V. Furthermore, adjusting for the number of close friends attenuates the effect of worsening health on suicide ideation. The number of close friends does not have an effect on suicide ideation in Wave IV. These results suggest that close friends become increasingly important for reducing the risk of suicide ideation throughout early adulthood.

**Table 4.** Weighted Estimates from Ordinary Least Squares Regression Models Predicting Number of Close Friends in Waves IV and V (*n* = 8,331).

	Wave IV		Wave V	
	Coefficient	95% CI	Coefficient	95% CI
Health status (reference = consistent)				
Worsened	−.11^+^	(−.22, .00)	−.18*	(−.32, −.04)
Improved	−.08	(−.25, .08)	.03	(−.12, .17)

*Source*: National Longitudinal Study of Adolescent to Adult Health (Add Health).

*Note*: Model adjusted for gender, race-ethnicity, foreign-born status, age, educational attainment, changes in employment status, changes in marital status, and changes in depression. CI = confidence interval.

* *p* < .05.

**Table 5.** Weighted Estimates (Odds Ratios) from Logistic Regression Models Predicting Suicide Ideation in Waves IV and V Controlling for Number of Close Friends (*n* = 8,331).

	Wave IV		Wave V	
	OR	95% CI	OR	95% CI
Health status (reference = consistent)				
Worsened	1.14	(.84, 1.55)	1.49**	(1.18, 1.87)
Improved	.89	(.60, 1.31)	.84	(.56, 1.24)
Number of close friends	1.00	(.93, 1.08)	.85***	(.78, .92)

*Source*: National Longitudinal Study of Adolescent to Adult Health (Add Health).

*Note*: Model adjusted for gender, race-ethnicity, foreign-born status, age, educational attainment, changes in employment status, changes in marital status, and changes in depression. OR = odds ratio; CI = confidence interval.

** *p* < .01, ***p < .001.

Then, we determine whether worsened health disrupts individuals’ roles and responsibilities, focusing on employment, parenthood, and marriage. Table 6 presents our main models stratified by employment status. Among those not employed at least 10 hours a week, worsened health does not significantly predict suicide ideation in either wave. Among those employed at least 10 hours a week, worsened health emerges as a significant predictor of suicide ideation in Wave V (OR = 1.88, 95% CI: 1.40 to 2.55), suggesting that health issues may increasingly disrupt responsibilities for the employed.

**Table 6.** Weighted Estimates (Odds Ratios) from Logistic Regression Models Predicting Suicide Ideation in Waves IV and V, Stratified by Employment Status.

	Not Employed ≥10 Hours/Week				Employed ≥10 Hours/Week			
	Wave IV		Wave V		Wave IV		Wave V	
	*n* = 1,360		*n* = 1,333		*n* = 6,971		*n* = 6,998	
	OR	95% CI	OR	95% CI	OR	95% CI	OR	95% CI
Health status (reference = consistent)								
Worsened	1.37	(.73, 2.54)	.84	(.45, 1.56)	1.08	(.75, 1.58)	1.88***	(1.40, 2.55)
Improved	.51	(.22, 1.19)	.57	(.23, 1.41)	1.08	(.70, 1.68)	.91	(.57, 1.44)

*Source*: National Longitudinal Study of Adolescent to Adult Health (Add Health).

*Note*: Model adjusted for gender, race-ethnicity, foreign-born status, age, educational attainment, changes in employment status, changes in marital status, and changes in depression. OR = odds ratio; CI = confidence interval.

*** *p* < .001.

Table 7 presents our main models stratified by the presence of children in the household. Among those with children, worsened health is not a significant predictor of suicide ideation in either wave. Among those without children, worsened health emerges as a larger and more significant predictor of suicide ideation in Wave V (OR = 1.70, 95% CI: 1.10 to 2.64) than in Wave IV (OR = 1.11, 95% CI: .75 to 1.66). Results in Appendix Table C1 in the online version of the journal indicate that the presence of children in the household is associated with lower odds of suicide ideation in both waves.

**Table 7.** Weighted Estimates (Odds Ratios) from Logistic Regression Models Predicting Suicide Ideation in Waves IV and V, Stratified by Presence of Children in the Household.

	No Children in the Household				Children in the Household			
	Wave IV		Wave V		Wave IV		Wave V	
	*n* = 4,445		*n* = 2,495		*n* = 3,886		*n* = 5,836	
	OR	95% CI	OR	95% CI	OR	95% CI	OR	95% CI
Health status (reference = consistent)								
Worsened	1.11	(.75, 1.66)	1.70*	(1.10, 2.64)	1.15	(.74, 1.80)	1.41	(.93, 2.13)
Improved	.98	(.60, 1.61)	.76	(.39, 1.50)	.71	(.36, 1.37)	.84	(.49, 1.43)

*Source*: National Longitudinal Study of Adolescent to Adult Health (Add Health).

*Note*: Model adjusted for gender, race-ethnicity, foreign-born status, age, educational attainment, changes in employment status, changes in marital status, and changes in depression. OR = odds ratio; CI = confidence interval.

* *p* < .05.

Table 8 presents our main results stratified by marital status. Among the unmarried, worsened health emerges as a significant predictor of suicide ideation in Wave V (OR = 1.44, 95% CI: 1.01 to 2.06). Among married individuals, worsened health predicts increased suicide ideation in both Wave IV (OR = 1.43, 95% CI: .91 to 2.25) and Wave V (OR = 1.57, 95% CI: .94 to 2.63), but sample sizes limit statistical power. Results in Appendix Tables D3 and D4 in the online version of the journal show that gender differences exist by marital status in Wave V, however, we cannot make reliable conclusions about these results due to power limitations.

**Table 8.** Weighted Estimates (Odds Ratios) from Logistic Regression Models Predicting Suicide Ideation in Waves IV and V, Stratified by Marital Status.

	Unmarried				Married			
	Wave IV		Wave V		Wave IV		Wave V	
	*n* = 4,522		*n* = 3,392		*n* = 3,809		*n* = 4,939	
	OR	95% CI	OR	95% CI	OR	95% CI	OR	95% CI
Health status (reference = consistent)								
Worsened	.97	(.63, 1.50)	1.44*	(1.01, 2.06)	1.43	(.91, 2.25)	1.57^+^	(.94, 2.63)
Improved	.89	(.56, 1.42)	.93	(.57, 1.51)	.82	(.41, 1.64)	.67	(.34, 1.34)

*Source*: National Longitudinal Study of Adolescent to Adult Health (Add Health).

*Note*: Model adjusted for gender, race-ethnicity, foreign born status, age, educational attainment, changes in employment status, changes in marital status, and changes in depression. OR = odds ratio; CI = confidence interval.

+ *p* < .1, *p < .05.

## Discussion

Our study examines the association between changes in health and suicide ideation throughout early adulthood, a life stage with shifts in household and union formation, labor force participation, and the nature of social relationships. Poor health contributes to social isolation (Hawthorne 2008) and the diminished capacity to maintain relationships (Haas et al. 2010) and impedes the ability to fulfill family responsibilities (Percheski and Meyer 2018) and maintain employment (Antonisse and Garfield 2018). Factors such as social isolation, unemployment or job loss, and divorce are established contributors to suicide ideation (Calati et al. 2019; Dalglish et al. 2015; Gratz et al. 2020; Kyung-Sook et al. 2018). Based on this, we anticipated that worsening health would likewise contribute to suicide ideation during early adulthood. We find in fully adjusted models that worsening health does not predict suicide ideation during emerging adulthood (ages 18–29) but appears as a significant predictor of suicide ideation during young adulthood (ages 30–45).

Our results confirm the importance of taking a life course approach to understanding the relationship between health and suicidality because the social determinants of health and suicide constantly shift in response to underlying social contexts that dominate different phases of the life course (Phillips and Hempstead 2022). Specifically, our results suggest that the importance of health as a determinant of suicidality grows with age in early adulthood as young adults begin to take on more adult roles and responsibilities and increasingly establish more permanent relationships.

We find that worsening health is more consequential for suicide ideation during young adulthood than emerging adulthood and test several possible explanations for this pattern. First, we find evidence that this pattern may in part reflect the increasing severity of health conditions. Our findings show that worsening health predicts more chronic conditions in young adulthood than emerging adulthood, which may indicate that reports of worsening health among those in their early 30s to early 40s reflect conditions that are more likely to disrupt day-to-day quality of life.

Second, we find evidence that this pattern may reflect the increasing importance of close relationships throughout early adulthood to suicide ideation, which can be disrupted by poor physical health. First, our results show that worsening health predicts a decrease in the number of close friends throughout early adulthood, confirming that poor health likely contributes to difficulty maintaining relationships (Haas, Schaefer, and Kornienko 2010). Second, while the number of close friends has no effect on suicide ideation in emerging adulthood, having fewer close friends significantly predicts suicide ideation in young adulthood. Young adults are immersed in larger and more permanent social networks than emerging adults (Harris and McDade 2018) and increasingly invested in the permanence and emotional returns of their relationships as they age (Sullivan-Singh et al. 2015), making them especially vulnerable to threats to these relationships (Shiner et al. 2009). Overall, our results suggest that worsening health contributes to difficulty maintaining close friendships, which may have a particularly robust effect on suicide ideation during young adulthood when close relationships are especially significant.

Of relevance to the sociological study of suicide, these results also contribute to the larger body of literature on the connection between social ties, health, and suicide. From a Durkheimian perspective, the relevance of social ties to suicide is fundamental because social relationships bring individuals meaning and purpose in their lives (Durkheim [1897] 1951; Mueller et al. 2021). From a neo-Durkheimian perspective, individual identities are rooted in social relationships, and subsequently, threats to social bonds and statuses are also salient threats to “social selves” (Abrutyn 2019). Thus, the more an individual is immersed in and attached to relationships or groups, the stronger the commitment and attachment is to a social identity, subsequently making the influence of others on the feelings and thoughts of the individual more robust (Mueller et al. 2021). In turn, commitment and attachment to others, and concomitant individual identities, render threats to social bonds because of the potential for exclusion, rejection, and isolation from others as well as loss of self (Abrutyn 2019; Mueller et al. 2021). From this neo-Durkheimian perspective, we argue that given emerging adulthood is typified by substantial identity exploration and often transient social relationships (Arnett 2012) and young adulthood represents a period with more solidified identities and relationships (Harris and McDade 2018; Shiner et al. 2009), threats to social relationships and auxiliary “social selves” become progressively more consequential for suicidality throughout early adulthood.

Furthermore, we find some evidence that the emergence of worsening health as a predictor of suicide ideation in young adulthood may reflect the age-graded nature of the acquirement of and immersion into social roles and responsibilities, explicitly in regard to employment. Our results indicate that worsening health as a significant predictor of suicide ideation is limited to those employed at least 10 hours a week in young adulthood. Furthermore, our main models show that while transitioning out of employment *reduces* the odds of suicide ideation in emerging adulthood, transitioning out of employment *increases* the odds of suicide ideation in young adulthood, which may imply that job loss is especially detrimental to mental health in young adulthood. Employment in emerging adulthood is often temporary and unstable (Arnett 2019), but by the early 30s, young adults are largely settled into more permanent employment with increasing role requirements because these jobs are likely ones they want to develop into long-term career paths (Arnett 2012, 2019). Poor health inhibits the ability to maintain employment (Antonisse and Garfield 2018) and fulfill job responsibilities (Percheski and Meyer 2018), and loss of employment and job insecurity are contributing facets to suicide ideation (Dalglish et al. 2015; Gratz et al. 2020). As such, our results support the notion that worsening health may affect the ability to fulfill job responsibilities, subsequently threatening employment, which may be especially consequential for suicide ideation among young adults who are increasingly invested in the development and permanence of their jobs.

However, contrary to our expectations, we do not find evidence that worsening health has a larger effect on suicide ideation among married adults and adults with children in the household. We suggest that this may reflect the protective nature of marriage and parenthood against suicide, which may offset difficulties maintaining marital and parenthood responsibilities due to poor health. For example, while poor health inhibits the ability to fulfill family responsibilities and contributes to union instability (Percheski and Meyer 2018), marriage and parenthood are two of the greatest protective agents against suicide (Dehara et al. 2021; Kyung-Sook et al. 2018). As such, having an intimate partner, such as a spouse, can provide emotional security and support and increase feelings of social integration (Caputo and Simon 2013), and having children can decrease feelings of loneliness (Dehara et al 2021). This is supported in our results that show that in young adulthood, those who were consistently married or transitioned into marriage had significantly lower odds of suicide ideation relative to those who remained unmarried (Table 3). Furthermore, among both emerging and young adults in our sample, having children in the household significantly reduces the odds of suicide ideation (Table C1 in the Appendix in the online version of the article). Overall, we suggest that it is possible that any harmful effects of poor health on the ability to fulfill family roles and responsibilities may be offset by the protective nature of marriage and parenthood and the social ties/integration they afford.

In our supplementary analyses, we tested for the possibility of gender differences in the effect of worsening health on suicide ideation. We might expect gender differences in these patterns given gendered processes of role expectations, social support, and health. We do not find gender differences in the overall effects, and gender differences by marital status do not tell a clear story. Moreover, we cannot ascertain concrete conclusions of the relationship between health and suicide ideation by gender and marital status due to our sample size and power limitations. Data permitting, future research should seek to disentangle these relationships.

There are several strengths to our study. Foremost, given the availability of longitudinal data, we were able to focus on *changes in health status* to investigate health trajectories as a predictor of suicide ideation in early adulthood. Similarly, the longitudinal nature of the survey facilitates examining the life course pattern of this relationship between ages 18 and 43. Second, our study utilized a robust set of controls, including mental health and life course transitions, that allowed us to hold these indicators constant in the relationship between changes in health status and suicide ideation.

Our study is not without limitations. First, Add Health does not directly ask respondents whether health affects the ability to fulfill roles and responsibilities across the waves, and thus, we were unable to fully account for this in our analyses. Second, our measure of health status is subjective. However, in a sensitivity analysis, we examined the relationship between changes in the number of chronic conditions and suicide ideation for each wave. In fully adjusted models (Appendix C in the online version of the article), we find that increases in the number of chronic health conditions had a significant effect on suicide ideation in Wave V but not Wave IV, which confirms the pattern of our results for changes in subjective health status. Third, we lack measures across all three waves of acute conditions. Ideally, we would have access to both acute and chronic conditions to account for the changing nature of health problems, but Add Health data are limited in this regard. Finally, there is a fair amount of attrition across the waves. We ran our main models with the inclusion of respondents lost to attrition in each wave and found that the results were substantively similar to the results of models using our sample (Appendix H1 in the online version of the article).

Our study provides a novel contribution to the literature on the relationship between health and suicidal thoughts by using a life course approach to contextualize the age-graded nature of this relationship. Our results underscore the importance of taking a life course approach because worsening health does not emerge as a significant predictor of suicide ideation until young adulthood. This implies that physical health ultimately becomes more important for mental well-being as one ages, and the reasons behind this are multifaceted. Our study has broader implications for social policies that can promote physical and mental well-being and reduce the likelihood of suicidal thoughts. These social policies may include improving access to physical and mental health resources through affordable health insurance and community health providers. Furthermore, social policies should focus on improving access to other resources that indirectly contribute to physical and mental health, such as affordable housing, education, and employment opportunities.

## Supplemental Material

sj-docx-1-hsb-10.1177_00221465221143768 – Supplemental material for Health, Suicidal Thoughts, and the Life Course: How Worsening Health Emerges as a Determinant of Suicide Ideation in Early Adulthood Click here for additional data file. Supplemental material, sj-docx-1-hsb-10.1177_00221465221143768 for Health, Suicidal Thoughts, and the Life Course: How Worsening Health Emerges as a Determinant of Suicide Ideation in Early Adulthood by Carlyn Graham and Andrew Fenelon in Journal of Health and Social Behavior
