# Supplementary material for: Health, Suicidal Thoughts, and the Life Course: How Worsening Health Emerges as a Determinant of Suicide Ideation in Early Adulthood
Source: J Health Soc Behav. 2023 Jan 12;64(1):62–78. doi: 10.1177/00221465221143768 (PMC10009325; doi:10.1177/00221465221143768)
Supplement: sj-docx-1-hsb-10.1177_00221465221143768 – Supplemental material for Health, Suicidal Thoughts, and the Life Course: How Worsening Health Emerges as a Determinant of Suicide Ideation in Early Adulthood [file sj-docx-1-hsb-10.1177_00221465221143768.docx]

**Journal** of **Health**

and **Social Behavior**

OFFICIAL JOURNAL OF THE AMERICAN SOCIOLOGICAL ASSOCIATION

**ONLINE SUPPLEMENT**

**to article in**

Journal of Health and Social Behavior

**Health, Suicidal Thoughts, and the Life Course: How Worsening Health Emerges as a Determinant of Suicide Ideation in Early Adulthood**

**Carlyn Graham**

*Pennsylvania State University*

**Andrew Fenelon**

*Pennsylvania State University*

APPENDIX A: Chronic conditions

Table A1. Weighted estimates (odds ratios) from logistic regression models predicting suicide ideation in Waves IV and V (n = 8,331)

|  | Wave IV | | Wave V | |
| --- | --- | --- | --- | --- |
|  | OR | 95% CI | OR | 95% CI |
| Chronic conditions (ref=consistent/decreased) |  |  |  |  |
| Increased | 1.09 | (0.79, 1.51) | 1.48* | (1.05, 2.09) |
| ***p<0.001 **p<0.01 *p<0.05 ^+^p<0.1 | |  |  |  |
| *Note: full model adjusted for gender, race/ethnicity, foreign born status, age, educational attainment, changes in employment status, changes in marital status and changes in depression* | | | | |
| *Source: National Longitudinal Study of Adolescent to Adult Health* | | | |  |

Table A2. Weighted estimates (odds ratios) from logistic regression models predicting suicide ideation in Waves IV and V controlling for number of chronic conditions (n = 8,331)

|  | Wave IV | | Wave V | |
| --- | --- | --- | --- | --- |
|  | OR | 95% CI | OR | 95% CI |
| Health status (ref=consistent) |  |  |  |  |
| Worsened | 1.12 | (0.82, 1.54) | 1.50** | (1.19, 1.90) |
| Improved | 0.89 | (0.60, 1.32) | 0.82 | (0.56, 1.22) |
| Number of chronic conditions | 1.11 | (0.93, 1.32) | 1.13^+^ | (0.98, 1.30) |
| ***p<0.001 **p<0.01 *p<0.05 ^+^p<0.1 | |  |  |  |
| *Note: full model adjusted for gender, race/ethnicity, foreign born status, age, educational attainment, changes in employment status, changes in marital status and changes in depression* | | | | |
| *Source: National Longitudinal Study of Adolescent to Adult Health* | | | |  |

APPENDIX B: Number of close friends

Table B1. Weighted estimates (odds ratios) from logistic regression models predicting suicide ideation in Waves IV and V, stratified by 0-2 close friends and 3 or more close friends

|  | 0-2 close friends | | | | 3 or more close friends | | | |
| --- | --- | --- | --- | --- | --- | --- | --- | --- |
|  | Wave IV | | Wave V | | Wave IV | | Wave V | |
|  | n = 1,959 | | n = 3,568 | | n = 6,372 | | n = 4,763 | |
|  | OR | 95% CI | OR | 95% CI | OR | 95% CI | OR | 95% CI |
| Health status (ref=consistent) |  |  |  |  |  |  |  |  |
| Worsened | 1.30 | (0.76, 2.23) | 1.37^+^ | (0.98, 1.92) | 1.09 | (0.76, 1.58) | 1.72* | (1.13, 2.61) |
| Improved | 0.71 | (0.31, 1.63) | 0.84 | (0.48, 1.45) | 0.98 | (0.64, 1.48) | 0.78 | (0.45, 1.36) |
| ***p<0.001 **p<0.01 *p<0.05 ^+^p<0.1 | | |  |  |  |  |  |  |
| *Note: full model adjusted for gender, race/ethnicity, foreign born status, age, educational attainment, changes in marital status, changes in employment status and changes in depression* | | | | | | | | |
| *Source: National Longitudinal Study of Adolescent to Adult Health* | | | | |  |  |  |  |

APPENDIX C: Social roles and responsibilities

Table C1. Weighted estimates (odds ratios) from logistic regression models predicting suicide ideation in Waves IV and V, controlling for presence of children in the household (n = 8,331)

|  | Wave IV | | Wave V | |
| --- | --- | --- | --- | --- |
|  | OR | 95% CI | OR | 95% CI |
| Health status (ref=consistent) |  |  |  |  |
| Worsened | 1.14 | (0.84, 1.54) | 1.53*** | (1.21, 1.93) |
| Improved | 0.87 | (0.59, 1.28) | 0.83 | (0.56, 1.22) |
| Presence of children in the household (ref=no) | 0.57** | (0.39, 0.82) | 0.61** | (0.46, 0.81) |
| ***p<0.001 **p<0.01 *p<0.05 ^+^p<0.1 |  |  |  |  |
| *Note: full model adjusted for gender, race/ethnicity, foreign born status, age, educational attainment, changes in employment status, changes in marital status and changes in depression* | | | | |
| *Source: National Longitudinal Study of Adolescent to Adult Health* | |  |  |  |

APPENDIX D: Gender differences

Table D1. Weighted estimates (odds ratios) from logistic regression models predicting suicide ideation in Waves IV and V, stratified by gender

|  | Men | | | | Women | | | |
| --- | --- | --- | --- | --- | --- | --- | --- | --- |
|  | Wave IV | | Wave V | | Wave IV | | Wave V | |
|  | n = 3,459 | | n = 3,459 | | n = 4,872 | | n =4,872 | |
|  | OR | 95% CI | OR | 95% CI | OR | 95% CI | OR | 95% CI |
| Health status (ref=consistent) |  |  |  |  |  |  |  |  |
| Worsened | 1.09 | (0.69, 1.74) | 1.47^+^ | (0.99, 2.17) | 1.19 | (0.81, 1.75) | 1.66** | (1.15, 2.39) |
| Improved | 0.89 | (0.43, 1.84) | 0.88 | (0.45, 1.74) | 0.93 | (0.59, 1.44) | 0.79 | (0.47, 1.33) |
| ***p<0.001 **p<0.01 *p<0.05 ^+^p<0.1 | | |  |  |  |  |  |  |
| *Note: full model adjusted for race/ethnicity, foreign born status, age, educational attainment, changes in employment status, changes in marital status and changes in depression* | | | | | | | | |
| *Source: National Longitudinal Study of Adolescent to Adult Health* | | | | |  |  |  |  |

Table D2. Weighted estimates (odds ratios) from logistic regression models predicting suicide ideation in Wave IV, stratified by gender and marital status

|  | Men | | | | Women | | | |
| --- | --- | --- | --- | --- | --- | --- | --- | --- |
|  | Unmarried | | Married | | Unmarried | | Married | |
|  | n = 1,977 | | n = 1,482 | | n = 2,545 | | n = 2,327 | |
|  | OR | 95% CI | OR | 95% CI | OR | 95% CI | OR | 95% CI |
| Health status (ref=consistent) |  |  |  |  |  |  |  |  |
| Worsened | 0.97 | (0.55, 1.73) | 1.35 | (0.61, 2.98) | 0.97 | (0.59, 1.61) | 1.59 | (0.87, 2.91) |
| Improved | 0.78 | (0.36, 1.71) | 1.27 | (0.36, 4.53) | 1.05 | (0.60, 1.86) | 0.59 | (0.23, 1.51) |
| ***p<0.001 **p<0.01 *p<0.05 +p<0.1 | | |  |  |  |  |  |  |
| *Note: full model adjusted for race/ethnicity, foreign born status, age, educational attainment, changes in employment status and changes in depression* | | | | | | | | |
| *Source: National Longitudinal Study of Adolescent to Adult Health* | | | | |  |  |  |  |

Table D3. Weighted estimates (odds ratios) from logistic regression models predicting suicide ideation in Wave V, stratified by gender and marital status

|  | Men | | | | Women | | | |
| --- | --- | --- | --- | --- | --- | --- | --- | --- |
|  | Unmarried | | Married | | Unmarried | | Married | |
|  | n = 1,389 | | n = 2,070 | | n = 2,003 | | n = 2,869 | |
|  | OR | 95% CI | OR | 95% CI | OR | 95% CI | OR | 95% CI |
| Health status (ref=consistent) |  |  |  |  |  |  |  |  |
| Worsened | 1.25 | (0.73, 2.15) | 2.14* | (1.08, 4.26) | 1.76* | (1.06, 2.91) | 1.34 | (0.71, 2.53) |
| Improved | 0.96 | (0.42, 2.23) | 0.96 | (0.34, 2.68) | 0.90 | (0.48, 1.70) | 0.53 | (0.18, 1.52) |
| ***p<0.001 **p<0.01 *p<0.05 +p<0.1 | | |  |  |  |  |  |  |
| *Note: full model adjusted for race/ethnicity, foreign born status, age, educational attainment, changes in employment status, changes in marital status and changes in depression* | | | | | | | | |
| *Source: National Longitudinal Study of Adolescent to Adult Health* | | | | |  |  |  |  |

APPENDIX E: Depression

Table E1. Weighted estimates (odds ratios) from logistic regression models predicting depression in Waves IV and V (n = 8,331)

|  | Wave IV | | Wave V | |
| --- | --- | --- | --- | --- |
|  | OR | 95% CI | OR | 95% CI |
| Health status (ref=consistent) |  |  |  |  |
| Worsened | 1.55*** | (1.29, 1.88) | 1.71*** | (1.41, 2.08) |
| Improved | 1.42* | (10.5, 1.92) | 1.02 | (0.73, 1.430 |
| ***p<0.001 **p<0.01 *p<0.05 +p<0.1 | |  |  |  |
| *Note: full model adjusted for gender, race/ethnicity, foreign born status, age, educational attainment, changes in employment status and changes in marital status* | | | | |
| *Source: National Longitudinal Study of Adolescent to Adult Health* | | |  |  |

APPENDIX F: Transition from adolescence (Wave II) to emerging adulthood (Wave III)

Table F1. Weighted estimates (odds ratios) from logistic regression models predicting suicide ideation in Wave III (n = 6,292)

|  | Model 1 | | Model 2 | | Model 3 | |
| --- | --- | --- | --- | --- | --- | --- |
|  | OR | 95% CI | OR | 95% CI | OR | 95% CI |
| Health status (ref=consistent) |  |  |  |  |  |  |
| Worsened | 1.76** | (1.20, 2.56) | 1.74** | (1.19, 2.53) | 1.35 | (0.91, 2.00) |
| Improved | 1.75** | (1.20, 2.55) | 1.80** | (1.22, 2.67) | 1.62* | (1.09, 2.39) |
| Female (ref=male) |  |  | 0.95 | (0.68, 1.34) | 0.86 | (0.61, 1.23) |
| Race/ethnicity (ref=non-Hispanic, White) |  |  |  |  |  |  |
| Hispanic |  |  | 0.76 | (0.50, 1.17) | 0.67 | (0.42, 1.06) |
| Non-Hispanic, Black |  |  | 0.53* | (0.31, 0.91) | 0.44** | (0.25, 0.79) |
| Non-Hispanic, Other |  |  | 0.90 | (0.53, 1.53) | 0.74 | (0.43, 1.25) |
| U.S. born (ref=foreign-born) |  |  | 0.84 | (0.44, 1.61) | 0.93 | (0.45, 1.92) |
| Age |  |  | 0.88* | (0.80, 0.97) | 0.92 | (0.82, 1.03) |
| Educational attainment (ref=less than high school) |  |  |  |  |  |  |
| High school or GED |  |  | 0.80 | (0.49, 1.31) | 0.94 | (0.56, 1.58) |
| Some college |  |  | 0.88 | (0.55, 1.39) | 1.13 | (0.70, 1.84) |
| 4-year degree or more |  |  | 0.99 | (0.52, 1.89) | 1.22 | (0.64, 2.31) |
| Employment status (ref=consistently not employed ≥10 hours/week) |  |  |  |  |  |  |
| Consistently employed ≥10 hours/week+ |  |  |  |  | 0.87 | (0.55, 1.36) |
| Transitioned into employment ≥10 hours/week |  |  |  |  | 0.90 | (0.52, 1.57) |
| Transitioned out of employment ≥10 hours/week |  |  |  |  | 1.13 | (0.81, 1.62) |
| Marital status (ref=consistently unmarried) |  |  |  |  |  |  |
| Consistently married |  |  |  |  | 0.39 | (0.05, 3.01) |
| Transitioned into marriage |  |  |  |  | 0.50* | (0.28, 0.90) |
| Transitioned out of marriage |  |  |  |  | 0.01*** | (0.002, 0.12) |
| Depression (ref=never depressed) |  |  |  |  |  |  |
| Consistently depressed |  |  |  |  | 7.39*** | (4.85, 11.28) |
| Depression onset |  |  |  |  | 4.42*** | (3.16, 6.17) |
| Depression recovery |  |  |  |  | 1.72^+^ | (0.95, 3.10) |
| ***p<0.001 **p<0.01 *p<0.05 ^+^p<0.1 |  |  |  |  |  |  |
| *Source: National Longitudinal Study of Adolescent to Adult Health* | | |  |  |  |  |

APPENDIX G. Pooled person-year data

Table G1. Weighted estimates (odds ratios) from logistic regression models predicting suicide ideation using pooled person-year data with and without the interaction of health status*age (n=26,665)

|  | Model 1 | | Model 2 | |
| --- | --- | --- | --- | --- |
|  | OR | 95% CI | OR | 95% CI |
| Health status (ref=excellent/very good) |  |  |  |  |
| Good | 1.37** | (1.15, 1.63) | 1.24 | (0.60, 2.56) |
| Fair/poor | 1.94*** | (1.55, 2.42) | 1.41 | (0.52, 3.84) |
| Female (ref=male) | 0.83+ | (0.68, 1.00) | 0.83^+^ | (0.68, 1.01) |
| Race/ethnicity (ref=non-Hispanic, White) |  |  |  |  |
| Hispanic | 0.76* | (0.60, 0.97) | 0.76* | (0.60, 0.97) |
| Non-Hispanic, Black | 0.56*** | (0.43, 0.72) | 0.56*** | (0.43, 0.72) |
| Non-Hispanic, Other | 0.88 | (0.64, 1.22) | 0.88 | (0.64, 1.22) |
| U.S. born (ref=foreign-born) | 0.85 | (0.57, 1.27) | 0.85 | (0.57, 1.27) |
| Age | 1.00 | (0.98, 1.01) | 0.99 | (0.97, 1.01) |
| Educational attainment (ref=less than high school) |  |  |  |  |
| High school or GED | 0.89 | (0.67, 1.18) | 0.89 | (0.67, 1.18) |
| Some college | 1.02 | (0.77, 1.36) | 1.02 | (0.77, 1.36) |
| 4-year degree or more | 0.89 | (0.67, 1.18) | 0.9 | (0.68, 1.18) |
| Not employed ≥10 hours/week (ref=employed ≥10 hours/week) | 1.23* | (1.02, 1.49) | 1.23* | (1.02, 1.48) |
| Married (ref=unmarried) | 0.58*** | (0.49, 0.68) | 0.58*** | (0.49, 0.69) |
| Depression (ref=not depressed) | 6.71*** | (5.83, 7.72) | 6.72*** | (5.84, 7.73) |
| Health status*age |  |  |  |  |
| Good |  |  | 1.00 | (0.98, 1.03) |
| Fair/poor |  |  | 1.01 | (0.98, 1.04) |
| ***p<0.001 **p<0.01 *p<0.05 ^+^p<0.1 |  |  |  |  |
| *Source: National Longitudinal Study of Adolescent to Adult Health* | | |  |  |

APPENDIX H: Attrition

Table H1. Weighted estimates (odds ratios) from logistic regression models predicting suicide ideation in Wave IV and V including respondents lost to attrition in each wave

|  | Wave IV | | Wave V | |
| --- | --- | --- | --- | --- |
|  | n = 11,827 | | n = 9,936 | |
|  | OR | 95% CI | OR | 95% CI |
| Health status (ref=consistent) |  |  |  |  |
| Worsened | 1.30* | (1.01, 1.66) | 1.51** | (1.20, 1.90) |
| Improved | 0.94 | (0.66, 1.32) | 0.82 | (0.53, 1.26) |
| ***p<0.001 **p<0.01 *p<0.05 ^+^p<0.1 | | |  |  |
| *Note: full model adjusted for gender, race/ethnicity, foreign born status, age, educational attainment, changes in employment status, changes in marital status and changes in depression* | | | | |
| *Source: National Longitudinal Study of Adolescent to Adult Health* | | | | |
